# An assessment of the characteristics and quality of diagnostic accuracy studies for positron emission tomography conducted in Japan: a systematic review

**DOI:** 10.1186/s13550-015-0084-4

**Published:** 2015-02-19

**Authors:** Shuhei Nomura, Akinori Hisashige, Daisuke Yoneoka, Mikiko Kanda, Karin Miyamoto, Miwako Segawa, Erika Ota, Kenji Shibuya

**Affiliations:** Department of Global Health Policy, Graduate School of Medicine, The University of Tokyo, 7-3-1 Hongo, Bunkyo-ku, Tokyo 113-0033 Japan; Institute of Healthcare Technology Assessment, 2-24-10, Shomachi, Tokushima, 770-0044 Japan; Present affiliation: Department of Epidemiology and Biostatistics, School of Public Health, Imperial College London, Norfolk Place, London, W2 1PG UK

**Keywords:** PET, Positron emission tomography, Japan, QUADAS

## Abstract

**Background:**

Systematic evaluations of the diagnostic accuracy of positron emission tomography (PET) imaging have been widely conducted in many countries. Although Japan’s total number of PET units is the second highest in the world, very limited effort has been made to systematically assess the methodological quality of PET studies in Japan. We performed a systematic review to assess the characteristics and quality of PET diagnostic accuracy studies conducted in Japan and to analyze the factors related to their quality.

**Methods:**

All PET studies conducted in Japan were identified using MEDLINE and the Japan Medical Abstract Society Database. The characteristics of the Japanese studies were examined and their methodological quality evaluated by the standardized quality assessment of diagnostic accuracy studies (QUADAS) tool. We compared the quality of studies indexed in MEDLINE with non-indexed studies, followed by a comparison of the studies’ conclusions with those of international health technology assessment (HTA) reports.

**Results:**

A total of 138 studies were identified. Half of them were not indexed in MEDLINE. The mean quality score of the Japanese studies was 6.7 and the proportion of high-quality studies (with a quality score higher than 8) was 32.6%. A significant difference was observed in several quality items between MEDLINE-indexed and non-indexed studies, although there was no difference in total quality score. Three variables (i.e., target diseases, publication year, and study type) were identified as factors related to the quality of the studies. Conclusions of Japanese studies relating to several target diseases were relatively consistent with international assessments.

**Conclusions:**

Although a considerable number of diagnostic accuracy studies of PET have been conducted in Japan, a substantial proportion of high-quality studies were not indexed in international databases. High-quality Japanese studies, therefore, should be searched using Japanese databases and assessed by systematic reviews and HTA conducted internationally.

**Electronic supplementary material:**

The online version of this article (doi:10.1186/s13550-015-0084-4) contains supplementary material, which is available to authorized users.

## Background

Positron emission tomography (PET) is a noninvasive imaging technique used for measuring the concentration of positron-emitting radioisotopes within tissue in malignant and benign disease and provides a three-dimensional image of functional changes in the body. PET can be used to assist management decisions related to diagnosis, staging/restaging, recurrence, and treatment planning and response as well as prognosis. Recently, an increasing number of clinical applications of PET have been observed, particularly in oncology, and mostly with the use of fluorine-18 fluorodeoxyglucose (18 F-FDG) as the PET tracer [1,2].

PET, however, is a high-cost technology, and it is therefore important for health policymakers to systematically assess not only the clinical indications of PET but also its cost-effectiveness in comparison with other competitive diagnostic technologies [3]. To this end, various countries have evaluated the efficacy and efficiency of PET since its introduction and diffusion into clinical practice [4-6].

In this evaluation, both diagnostic accuracy and methodological quality of diagnostic studies are important elements of this evaluation. However, diagnostic studies have several unique features in terms of quality, which are not addressed by the traditional approach to evaluating controlled trials. A validated quality assessment tool is currently not available [7]. In 2003, the Standards for the Reporting of Diagnostic Accuracy (STARD) statement was developed to help authors improve reporting [8], and the quality assessment of diagnostic accuracy studies (QUADAS) tool was developed in the same year [9]. Despite these advancements and the development of study selection tools for systematic reviews [4,6,10,11], comprehensive quality assessment of PET diagnostic studies has been very limited.

In Japan, while PET has been introduced and diffused without a systematic health technology assessment, the total number (i.e., 466) of PET units installed in Japan was the second highest in the world following the United States (i.e., 1,450) in 2011 [12]. A considerable number of diagnostic studies for PET have been conducted, but as they are primarily published in Japanese, they have not been widely reported in international journals and databases.

Therefore, even though Japanese studies have made a contribution to the evaluation of diagnostic accuracy of PET both in Japan and internationally, comprehensive information about PET studies conducted in Japan is greatly lacking. We conducted a systematic review to assess the characteristics and quality of PET studies in Japan and analyze the factors related to their quality.

## Methods

### Search strategy

All papers reporting diagnostic efficacy studies for PET conducted in Japan as original articles were identified through two databases: the international database, MEDLINE, and Ichu-Shi, the domestic Japanese database of the Japan Medical Abstract Society. MEDLINE includes Japanese papers written in English, as well as only English summaries for a very limited number of papers written in Japanese. We searched this database from inception to 15 July 2011. Ichu-Shi includes studies written in Japanese and published in Japanese journals and occasionally features an English summary. We searched this database from inception to 23 August 2011. The search strategy used the following general terms, expanded and appropriately modified for each database: ‘positron emission tomography’ or ‘positron emission computed tomography’ or ‘PET’, and ‘sensitivity’ and ‘specificity’. The search terms are presented in Additional file 1: Table S1 and Additional file 2: Table S2.

### Inclusion and exclusion criteria

All papers reporting diagnostic efficacy or accuracy studies of PET conducted in Japan that used data from PET scans performed at institutions in Japan and were published as original articles until the end of 2010 were included. The following types of PET studies were excluded: 1) non-diagnostic studies, such as studies of treatment planning, and response and prognosis; 2) studies that did not explicitly describe sensitivity and/or specificity of PET, and which could not be derived from the data provided in the paper; 3) studies written in languages other than Japanese or English, and 4) case reports, systematic reviews and meta-analyses.

### Study selection and data extraction

Two reviewers independently screened titles and abstracts of studies identified by the search. The text of all potentially relevant studies was evaluated in detail and assessed against eligibility criteria. The following data were extracted and checked independently by two reviewers: database indexed, publication year, index test, study type, sample size, target disease, comparator, study subjects, outcome (i.e., sensitivity and specificity), conclusions, and funding source. Any disagreements were resolved by consensus between the reviewers.

### Assessment of quality of studies

The QUADAS tool was used to assess the methodological quality of studies. This tool is a validated quality checklist containing 14 items that address the most important sources of bias and variation in diagnostic accuracy studies [7,9,13]. QUADAS was also adopted by the Cochrane Collaboration in their handbook of diagnostic systematic reviews [14]. The detailed explanations of the 14 items were listed in Additional file 3: Table S3.

The reviewers who assessed and extracted data were trained in the use of the QUADAS checklist. Each item in the checklist was categorized as ‘Yes’ for low risk of bias, ‘No’ for high risk of bias, or ‘Unclear’ if there was insufficient information to make a judgment. We also calculated a quality score defined as the total number of items categorized as ‘Yes’ among 14 items.

### Data analysis

Firstly, we identified the number of diagnostic efficacy studies of PET conducted in Japan from 1990 to 2010 and examined their characteristics. Then we assessed the quality of these studies using the QUADAS tool. Secondly, we evaluated the quality of these studies by comparing studies indexed in MEDLINE with studies published in Japanese journals that were not indexed in international databases. We used Fisher’s exact test for proportions and *t*-test for quality scores in the statistical analysis.

Thirdly, the factors affecting the quality of Japanese PET studies were analyzed using a multiple logistic regression model. The dependent variable was whether the quality of the study was high (=1) or low (=0) [15-17]. We defined studies as high quality when the quality score exceeded eight (i.e., more than a half of total score), based on a definition from a previous systematic review [18]. The independent variables were target disease, publication year, sample size, study design, funding source, international indexing, and whether or not comparative statistical analysis was conducted. All analyses were performed with STATA/MP 13.

Finally, the results and conclusions of the Japanese studies were compared with those of international HTA reports and systematic reviews in clinical medicine, in relation to PET and other competitive imaging technologies. We made this comparison to primarily examine the correspondence of the results and conclusions of Japanese studies with those of the international assessments. In the comparison, instead of an integrated form, only a proportion of positive conclusions, as well as a quality score and a proportion of comparative studies, are presented for the reader’s own consideration since there is no explicit or standardized guideline for integrating conclusions or recommendations of accumulated studies. In addition, we assessed the coverage of different diseases among both the Japanese studies and international assessments to examine whether Japanese studies covered some diseases that international assessments did not. HTA studies related to PET were comprehensively identified by database searches (i.e., the Centre for Reviews and Dissemination (CRD) database, a health services research center based at the University of York). Of the 49 reports identified and retrieved, two Belgian Health Care Knowledge Centre (KCE) reports were selected as a reference [6,10], which are the latest and most comprehensive assessments of high quality according to the INAHTA checklist for HTA reports [19]. We found only one systematic review that comprehensively covered areas of disease [11]. Note that as the number of Japanese studies was limited, we analyzed target diseases that were examined in more than three of the Japanese studies.

## Results

### Search results

**Figure 1:**
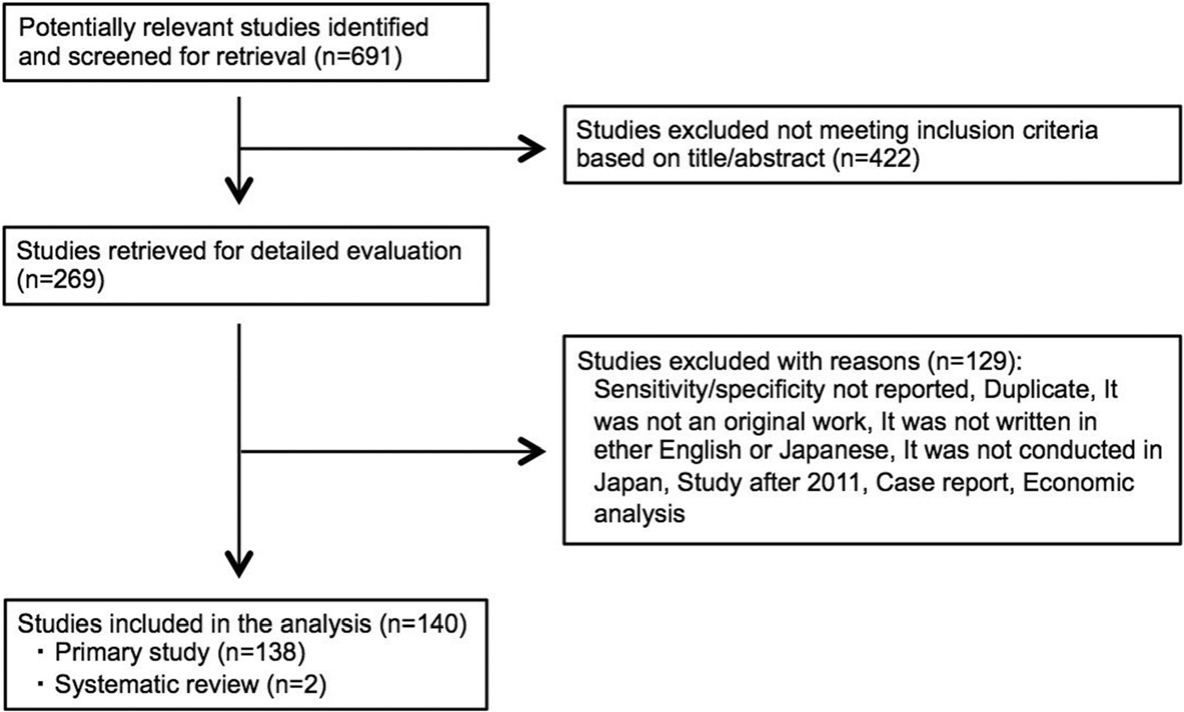
**Flow diagram for study selection.**

A total of 691 potentially relevant studies were identified from database searches (Figure 1). Of these, 269 studies were evaluated in detail. Finally, a total of 138 studies were determined to meet eligibility criteria and included in the analysis. Of the total of 138 included studies, 70 studies were written in Japanese and 68 studies were written in English, of which 3 studies and 66 studies were indexed in MEDLINE, respectively.

### Characteristics of Japanese studies

Nearly 90% of the studies evaluated malignant neoplasms and mostly examined lung cancer (21.7%), followed by head and neck cancer (8.7%) and breast cancer (7.2%), respectively (Table 1). Of the 39 articles classified into ‘others’, 10 (7.2%) investigated non-malignant diseases or disorders, such as gallbladder wall thickening, artery occlusive diseases, aortic graft infection, and pneumoconiosis. Ninety-six percent of the studies used 18 F-FDG as a PET tracer. More than half of the studies were published after 2007. The sample size among 57.9% of the studies was less than 50. Only one study employed more than 500 patients. Comparative analyses with other competitive diagnostic technologies, such as MRI and/or CT, were conducted in 47.8% of the studies. The most frequent type of study design was the retrospective study (74.6%). Funding sources were not reported in 88% of the studies.

**Table 1:** **Characteristics of included studies by year of application/revision of the insurance service**

**Characteristics**	**Number of articles (%)**
Diseases	
Lung cancer	30 (21.7)
Lymphoma	5 (3.6)
Head and neck cancer	12 (8.7)
Colorectal cancer	8 (5.8)
Breast cancer	10 (7.2)
Esophageal cancer	4 (2.9)
Stomach cancer	4 (2.9)
Thyroid cancer	1 (0.7)
Pancreatic cancer	6 (4.3)
Cervical cancer	4 (2.9)
Ovarian cancer	4 (2.9)
Uterine cancer	6 (4.3)
Prostate cancer	2 (1.4)
Gastrointestinal stromal tumor	1 (0.7)
Brain cancer	1 (0.7)
Alzheimer’s disease	1 (0.7)
Others	39 (28.2)
Modality	
PET	94 (68.1)
PET/CT	48 (34.8)
PET tracer	
18 F-FDG	133 (96.4)
Others	5 (3.6)
Publication year	
2002	14 (10.1)
2003 to 2006	38 (27.5)
2007 to 2010	86 (62.3)
Sample size	
Less than 24	30 (21.7)
25 to 49	50 (36.2)
50 to 99	31 (22.5)
More than 100	27 (19.5)
Study type	
Comparative study	68 (49.3)
Non-comparative study	70 (50.7)
Study design	
Prospective study	35 (25.4)
Retrospective study	103 (74.6)
Funding source	
Description included	17 (12.3)
No description	121 (87.7)
Index type	
Indexed in MEDLINE	69 (50.0)
Not indexed in MEDLINE	69 (50.0)

### Methodological quality of Japanese studies

Studies were assessed using QUADAS (Additional file 3: Table S3). The proportion of studies with high quality, in which eight or more items were categorized as ‘Yes’ for low risk of bias, was 32.6%. The mean (SD) quality score out of 14 items was 6.7 (2.5).

**Figure 2:**
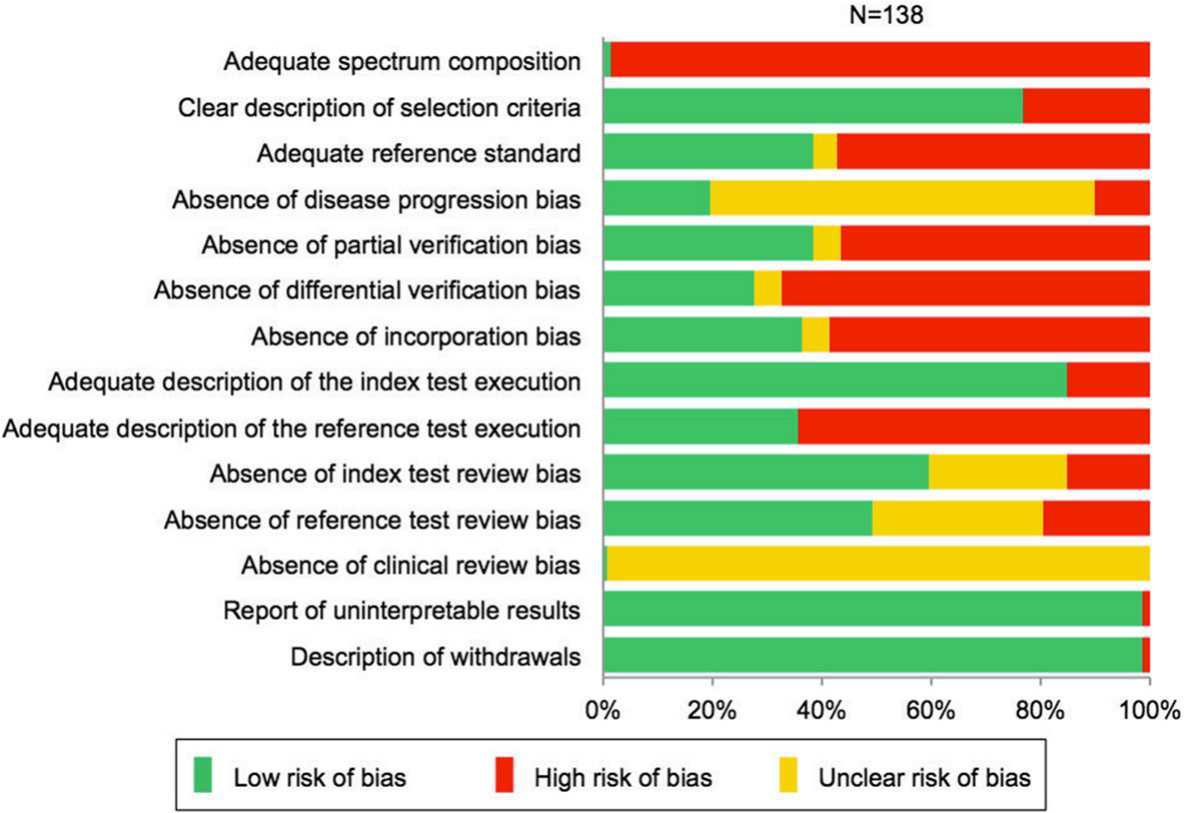
**Quality of diagnostic studies assessed by QUADAS.**

As shown in Figure 2, the following 6 of 14 items were observed to have high risk of bias, where the proportion of items categorized as ‘No’ was high (more than 50%): adequate spectrum (item 1), adequate reference standard (item 3), partial verification (item 5), differential verification (item 6), incorporation bias (item 7), and description of reference test execution (item 9). Low risk of bias, where the proportion of items categorized as ‘Yes’ was high (more than 50%), was observed in the following five items: selection criteria (item 2), description of the index test (item 8), index test review (item 10), uninterpretable results (item 13), and withdrawals (item 14). The following two items were categorized as ‘Unclear’ due to a lack of sufficient information to make a judgment: disease progression (item 4) and clinical review (item 12).

### Comparison between MEDLINE-indexed and non-indexed studies

**Figure 3:**
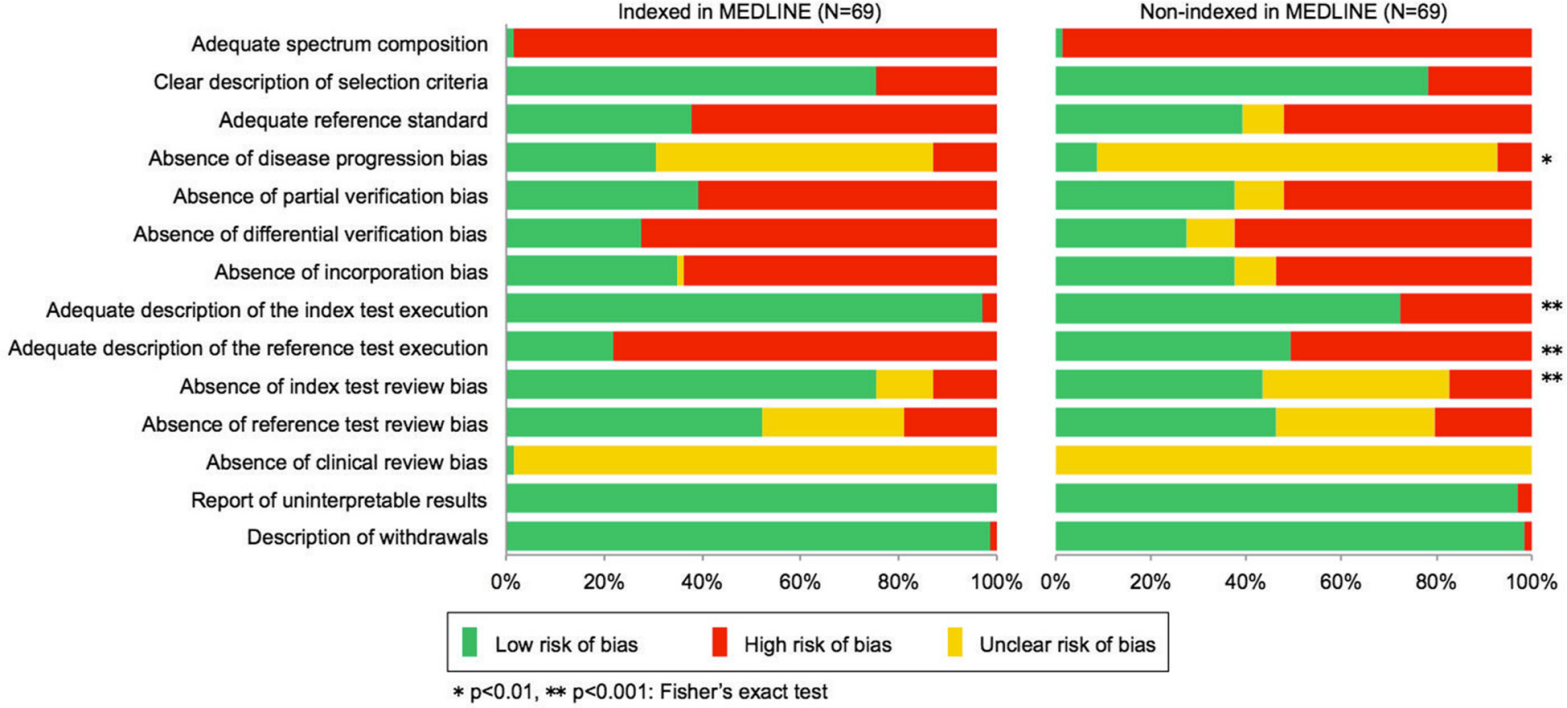
**Comparison of quality between MEDLINE-indexed and non-indexed studies.**

Figure 3 compares the results in quality between the MEDLINE-indexed and non-indexed studies. The proportion of items categorized as ‘Yes’ among MEDLINE-indexed studies was significantly higher than among non-indexed studies in disease progression, (item 4) (*p* = 0.009, 30.4% for indexed studies and 8.7% for non-indexed studies); description of index test execution (item 8) (*p* < 0.001, 97.1% for indexed studies and 72.5% for non-indexed studies); and index test review (item 10) (*p* < 0.001, 75.4% for indexed studies and 43.5% for non-indexed studies). In contrast, we observed a lower proportion of ‘Yes’ items in MEDLINE-indexed studies for the description of reference test (item 9) (*p* < 0.001, 21.7% for indexed studies and 49.3% for non-indexed studies). However, there is no statistical difference in the mean quality score between them (*p* = 0.3). Note that as the language type of the paper closely corresponded to whether or not the study was indexed in MEDLINE, the differences in methodological quality between the English and Japanese papers were similar to those observed between papers indexed in MEDLINE and those that were not (data are not shown).

### Factors related to the quality of studies

Table 2 shows the result of a multiple logistic regression analysis to examine the factors related to the methodological quality. Of all seven factors employed, target disease, publication year, and study design were shown to be associated with study quality. Target diseases other than respiratory cancer, gastrointestinal cancer, gynecological cancer, head and neck cancer, and breast cancer, which were categorized as ‘others’, were lower in quality (*p* = 0.02). Studies published between 2003 and 2006 were lower in quality than those published before 2002 (*p* = 0.01). The methodological quality of studies between 2007 and 2010 was also lower than that before 2002, but the difference was not statistically significant (*p* = 0.1). Also, prospective studies were higher in quality than retrospective studies (*p* = 0.04).

**Table 2:** **Factors related to methodological quality**

**Variables**	**Number of studies**	**Odds ratio**	**95% confidence interval**	***p*** **value**
Diseases				
Respiratory cancer	30	Ref.		
Gastrointestinal cancer	7	0.98	0.13 to 7.44	1.0
Gynecological cancer	19	0.38	0.09 to 1.56	0.2
Head and neck cancer	14	0.39	0.08 to 1.80	0.2
Breast cancer	10	0.23	0.04 to 1.46	0.1
Others	58	0.23	0.07 to 0.76	0.02
Publication year				
2002	14	Ref.		
2003 to 2006	38	0.11	0.02 to 0.53	0.01
2007 to 2010	86	0.30	0.07 to 1.30	0.1
Sample size				
Less than 24	30	Ref.		
25 to 49	50	1.43	0.47 to 4.35	0.5
50 to 99	31	1.00	0.28 to 3.58	1.0
Over 100	27	0.53	0.13 to 2.12	0.4
Study design				
Retrospective study	103	Ref.		
Prospective study	35	2.78	1.05 to 7.35	0.04
Funding source				
No description	121	Ref.		
Description included	17	2.9	0.78 to 10.73	0.1
Index type				
Not indexed in MEDLINE	69	Ref.		
Indexed in MEDLINE	69	0.84	0.34 to 2.10	0.7
Comparative study				
No	72	Ref.		
Yes	66	1.53	0.64 to 3.62	0.3

### Comparing the conclusions of Japanese studies and international HTA reports and systematic reviews

Table 3 shows a comparison between the conclusions of Japanese studies with international HTA reports and the systematic review in oncology. While the conclusions in breast, esophageal, and ovarian cancers were relatively consistent among Japanese studies and international assessments, those in other target diseases, including lung cancer, lymphoma, and head and neck cancer, were inconsistent among them.

**Table 3:** **Conclusions of Japanese studies compared with those of international HTA reports**

**Diseases**	**Number of studies**	**Mean quality score**	**Comparative study (%)**	Positive conclusion* (%)	**International HTA report**	**Recommendation of PET use in oncology**
Lung cancer	30	7.2	11 (37)	8 (27)	○ (SNP, NCLC)	Yes (SNP, NCLC)
Lymphoma	5	6	4 (80)	3 (60)	×	No
Head and neck cancer	12	6.7	5 (42)	5 (42)	○	Yes
Colorectal cancer	8	6.6	3 (38)	4 (50)	×	No
Breast cancer	10	5.7	4 (40)	3 (30)	×	No
Esophageal cancer	4	8.8	3 (75)	1 (25)	Lack of evidence	No
Stomach cancer	4	5.8	2 (50)	3 (75)	Lack of evidence	-
Pancreatic cancer	6	8.7	4 (67)	4 (67)	Unclear	Yes
Cervical cancer	4	7.5	3 (75)	3 (75)	Lack of evidence	-
Ovarian cancer	4	6.3	4 (100)	3 (75)	○	-
Uterine cancer	6	7.8	3 (50)	0 (0)	Lack of evidence	-

*Positive: PET(/CT) was superior to other competitive diagnostic technologies.

○, evidence for diagnostic accuracy; ×, non-evidence for diagnostic accuracy; SNP, solitary pulmonary nodule; NCLC, non-small-cell lung cancer.

In addition, the Japanese studies covered many other areas of disease (e.g., stomach, cervical, and uterine cancers) where evidence and recommendations were lacking in the international assessments. The number of studies in Japan varied widely from 4 to 30 depending on disease areas, and quality scores ranged from 5.7 to 8.8. The proportion of comparative studies also varied widely from 38% to 100%, and the proportion of positive conclusions ranged from 0% to 75%. The number and quality scores of studies, and the proportion of comparative studies, were not necessarily correlated to the proportion of positive results and the discrepancies among conclusions.

## Discussion

This study is the first systematic review of the characteristics and quality of diagnostic accuracy studies of PET conducted in Japan. Although a total of 138 Japanese studies involving PET were identified, half of them were not indexed in MEDLINE. Although a potential overlap of study subjects may exist among several studies, this could not be taken into consideration due to a lack of information about the study participants in some studies. Also, papers with different aims and methods were considered as independent studies. In contrast, the total number of studies reviewed in a HTA report in the UK was 158 and included 6 non-English studies indexed in international databases [4]. Also, languages in the primary study selection were limited to several European languages in the Belgian report [6,10]. Therefore, non-indexed Japanese studies or studies written in Japanese would likely be missed from international HTA reports.

Malignant neoplasm was the target disease most frequently covered by Japanese studies (Table 1). This is a similar finding to previous international studies [4,10]. Fifty-eight percent of Japanese studies had a sample size less than 50. The estimates of accuracy in small studies are often inexact and their results have little generalizability for target patients [20]. Also, Bachmann et al. estimated that the median number of patients with or without a target condition necessary to calculate valid sensitivity and specificity of diagnostic accuracy is 49 and 76, respectively. The sample size for most international PET studies was also less than 100 [4,6,10]. In addition, approximately 90% of Japanese studies did not include information about funding sources. A systematic review of conflict of interests highlighted that systematic biases support products created by the funder [21], thus implying that hidden conflicts of interest may be present among the Japanese studies.

Our study showed that the mean quality score was 6.7 (e.g., a full score is 14), and 33% of Japanese PET studies were of high quality, as indicated by the quality score of more than 8. These results were similar to those of several recent systematic reviews [22,23]. Also, a high risk of bias was observed in six items including adequate spectrum, adequate reference standard, and absence of verification bias, among others (Figure 2). This result indicates that the Japanese studies have numerous biases and are of relatively low quality, which is limitedly applicable to PET use in clinical settings. For example, PET studies of low quality were excluded from international health technology assessments (i.e., quality score less than eight) [18], or critically examined in clinical recommendations [4,6,10]. Therefore, studies of low quality will neither be used nor reflected in clinical guidelines and health policies. Moreover, the quality of test studies is extremely important as a basis for further evaluation for clinical decision making and health outcomes [24,25]. Greater improvement of the quality of test studies is urgently needed.

Factors related to the methodological quality, target disease, publication year, and study design were determined by multiple logistic regression analysis (Table 2). Prior to 2002 when PET examination was first included in the National Health Insurance, the quality of studies was high. This may be because that prior to the application of insurance coverage, PET researchers, related academies and industries proactively and rigorously conducted and published many PET studies to encourage and persuade the government to include PET testing in the insurance scheme, as well as to promote the utilization of PET testing after its inclusion in the insurance coverage. These efforts to promote PET testing seem to have had a positive influence on maintaining the methodological quality of the studies and overcoming critical assessments from the government. As PET studies have been conducted mainly in the area of oncology, particularly in respiratory cancer, the quality of studies based on target disease will improve over time as research applications of PET expand into other areas. Our results are consistent with previous research which highlighted that prospective studies are favorable for reducing biases [26].

Our assessment of the characteristics and quality of Japanese PET studies demonstrates that efforts to educate researchers, provide incentives, and establish systems for conducting diagnostic studies are needed to encourage investigators to comply with existing methodological standards. Low quality of reporting was found to be a significant obstacle in the evaluation of quality, and therefore the risk of bias remained unclarified in this study. As many studies have limited applicability in clinical practice and health policy, their inclusion might be misleading in some cases. In this study, a high proportion of ‘unclear’ results were observed in several items of risk of bias (Figure 2), which proved difficult for reviewers to evaluate the actual quality of the studies. This result is also reported in several systematic reviews [18,19]. Concerns about the quality of reporting of diagnostic studies led to the endorsement of the STARD statement [27]. Since the publication of this statement, the quality of reporting of diagnostic accuracy studies has slightly improved [28]. To advance the quality of reporting in Japan, efforts are required to raise awareness of the STARD statement and to encourage publishers of Japanese scientific journals to adopt the statement in their instructions to authors.

Conversely, 34.9% of studies not indexed in MEDLINE and 30.4% of the indexed studies were of high quality with a quality score of eight or higher. There was no significant difference in the total quality score between the two groups, even though a significant difference was observed in several items between them (Figure 3). After adjusting for other factors in a logistic regression model, the overall quality was not significantly different between indexed PET studies and those not indexed in MEDLINE (Table 2). This result suggests that non-indexed Japanese studies should be included in systematic reviews as well as both international and Japanese databases in order to prevent the exclusion of high-quality Japanese PET studies. In addition, excluding Japanese studies may introduce a language bias and lead to erroneous conclusions.

The search and collection of non-English language papers is important to minimize language bias [29]. In conducting systematic reviews, international collaboration in the area where language bias might occur could be a practical and feasible solution for minimizing language bias. On the other hand, non-English-speaking researchers should also be encouraged to publish original studies in English in a journal indexed in international databases.

Finally, only 47.8% of Japanese studies employed comparators (i.e., competitive diagnostic technologies such as MRI or CT) to evaluate diagnostic accuracy of PET. Of this percentage, only 23 studies performed statistical analysis. As the diagnostic accuracy of non-comparative studies often differs to that of comparative studies [30], the conclusions of the Japanese studies should be carefully interpreted. However, there has been no mention of this issue even in systematic reviews and HTA reports of PET studies [4,22,23,30]. In addition, only 6.9% of non-comparative studies performed simple comparisons with the results obtained from literature surveys.

These issues might influence the discrepancy of conclusions between Japanese studies and international assessments. For discrepancies found in the coverage of disease areas, Japanese studies could serve as supplementary information for the conclusions or recommendations of international assessments to prevent language bias, since international assessments do not include most Japanese studies. However, further systematic examination would be needed to integrate the information and assess the influence on conclusions and recommendations, since there is no explicit or standardized guideline for integrating these conclusions or recommendations.

On the other hand, in regard to the disease areas where only Japanese studies were available, the application of the Japanese National Health Insurance was based on a small number of studies with relatively low quality scores. In the case of uterine cancer, there is no positive conclusion. In Japan, since there has been neither comprehensive HTA nor guidance based on systematic reviews, further examinations would be required for health and clinical policy.

## Conclusions

The Japanese studies covered a wide range of target diseases, which were not evaluated by the HTA and the systematic review. For a practical solution of these issues, Japanese studies or other language studies should be comprehensively included and simultaneously evaluated when conducting HTA or systematic reviews.

Diverse factors such as study design, conduct, analysis, and reporting of PET studies are related to the quality, number, and progress of PET studies in Japan. Greater efforts are required to set and implement feasible strategies for improving Japanese PET studies under the collective action of all stakeholders.

## Additional files

Additional file 1: Table S1. Search strategy for article selection on Ovid MEDLINE(R) In-Process & Other Non-Indexed Citations, Ovid MEDLINE(R) and Ovid OLDMEDLINE(R) 1946 to Present with Daily Update. Additional file 2: Table S2. Search strategy for article selection on Ichushi web. Additional file 3: Table S3. Detailed descriptions of QUADAS items.

## Abbreviations

HTA: health technology assessment
PET: positron emission tomography
QUADAS: quality assessment of diagnostic accuracy studies
