# Supplementary material for: An assessment of the characteristics and quality of diagnostic accuracy studies for positron emission tomography conducted in Japan: a systematic review
Source: EJNMMI Res. 2015 Feb 19;5:6. doi: 10.1186/s13550-015-0084-4 (PMC4385095; doi:10.1186/s13550-015-0084-4)
Supplement: Additional file 1: Table S1. — Search strategy for article selection on Ovid MEDLINE(R) In-Process & Other Non-Indexed Citations, Ovid MEDLINE(R) and Ovid OLDMEDLINE(R) 1946 to Present with Daily Update. [file 13550_2015_84_MOESM1_ESM.docx]

Additional file 1: Table S1: Search strategy for article selection on Ovid MEDLINE(R) In-Process & Other Non-Indexed Citations, Ovid MEDLINE(R) and Ovid OLDMEDLINE(R) 1946 to Present with Daily Update

| Step | Search term |
| --- | --- |
| 1 | Positron-Emission Tomography/ |
| 2 | positron emission tomography.tw. |
| 3 | positron emission computerized tomography.tw. |
| 4 | PET.tw. |
| 5 | or/1-4 |
| 6 | Sensitivity and Specificity/ |
| 7 | Sensitivity.tw. |
| 8 | specificity.tw. |
| 9 | or/6-8 |
| 10 | 5 and 9 |
| 11 | japan/ |
| 12 | japan$.tw. |
| 13 | or/11-12 |
| 14 | 10 and 13 |
| 15 | limit 14 to humans |
| 16 | limit 15 to abstracts |
| 17 | Review.pt. |
| 18 | 16 not 17 |
