# Supplementary material for: An assessment of the characteristics and quality of diagnostic accuracy studies for positron emission tomography conducted in Japan: a systematic review
Source: EJNMMI Res. 2015 Feb 19;5:6. doi: 10.1186/s13550-015-0084-4 (PMC4385095; doi:10.1186/s13550-015-0084-4)
Supplement: Additional file 2: Table S2. — Search strategy for article selection on Ichushi web. [file 13550_2015_84_MOESM2_ESM.doc]

Additional file 2: Table S2 Search strategy for article selection on Ichushi web

| Step | Search term |
| --- | --- |
| 1 | 陽電子放射型断層撮影/TH or PET/AL or "Positron Emission Tomography"/AL or 陽電子断層/AL or 陽電子放出断層/AL or 陽電子放出型断層/AL or 陽電子放射断層/AL or 陽電子放出CT/AL or 陽電子放出トモグラフィー/AL or 陽電子放射断層/AL or 陽電子射出断層/AL or 陽電子画像/AL or ポジトロン断層/AL or ポジトロン放出断層/AL or ポジトロン放出型断層/AL or ポジトロン放射断層/AL or ポジトロンCT/AL or ポジトロンエミッショントモグラフィー/AL or ポジトロン・エミッション・トモグラフィー/AL or ポジトロン核医学/AL |
| 2 | 感度と特異度/TH or 感度/AL or Sensitivity/AL or 特異度/AL or Specificity/AL or 敏感度/AL or 感受性/AL or 特異性/AL |
| 3 | #1 and #2 |
| 4 | (#3) and (AB=Y) |
| 5 | (#3) and (AB=Y CK=ヒト) |
