# Supplementary material for: An assessment of the characteristics and quality of diagnostic accuracy studies for positron emission tomography conducted in Japan: a systematic review
Source: EJNMMI Res. 2015 Feb 19;5:6. doi: 10.1186/s13550-015-0084-4 (PMC4385095; doi:10.1186/s13550-015-0084-4)
Supplement: Additional file 3: Table S3. — Detailed descriptions of QUADAS items. [file 13550_2015_84_MOESM3_ESM.docx]

Additional file 3: Table S3: Detailed descriptions of QUADAS items

| Item number | Question | What is meant by this item |
| --- | --- | --- |
| 1 | Was the spectrum of patients representative of the patients who will receive the test in practice? | Differences in demographic and clinical features between populations may produce measures of diagnostic accuracy that vary considerably, this is known as spectrum bias. It refers more to the generalisability of results than to the possibility that the study may produce biased results. Reported estimates of diagnostic accuracy may have limited clinical applicability (generalisability) if the spectrum of tested patients is not similar to the patients in whom the test will be used in practice. The spectrum of patients refers not only to the severity of the underlying target condition, but also to demographic features and to the presence of differential diagnosis and/or co-morbidity. It is therefore important that diagnostic test evaluations include an appropriate spectrum of patients for the test under investigation and also that a clear description is provided of the population actually included in the study. |
| 2 | Were selection criteria clearly described? | This refers to whether studies have provided a clear definition of the criteria used as in- and exclusion criteria for entry into the study. |
| 3 | Is the reference standard likely to correctly classify the target condition? | The reference standard is the method used to determine the presence or absence of the target condition. To assess the diagnostic accuracy of the index test its results are compared with the results of the reference standard; subsequently indicators of diagnostic accuracy can be calculated. The reference standard is therefore an important determinant of the diagnostic accuracy of a test. Estimates of test performance are based on the assumption that the index test is being compared to a reference standard which is 100% sensitive and specific. If there are any disagreements between the reference standard and the index test then it is assumed that the index test is incorrect. Thus, from a theoretical point of view the choice of an appropriate reference standard is very important. |
| 4 | Is the time period between reference standard and index test short enough to be reasonably sure that the target condition did not change between the two tests? | Ideally the results of the index test and the reference standard are collected on the same patients at the same time. If this is not possible and a delay occurs, misclassification due to spontaneous recovery or to progression to a more advanced stage of disease may occur. This is known as disease progression bias. The length of the time period which may cause such bias will vary between conditions. For example a delay of a few days is unlikely to be a problem for chronic conditions, however, for many infectious diseases a delay between performance of index and reference standard of only a few days may be important. This type of bias may occur in chronic conditions in which the reference standard involves clinical follow-up of several years. |
| 5 | Did the whole sample or a random selection of the sample, receive verification using a reference standard of diagnosis? | Partial verification bias (also known as work-up bias, (primary) selection bias, or sequential ordering bias) occurs when not all of the study group receive confirmation of the diagnosis by the reference standard. If the results of the index test influence the decision to perform the reference standard then biased estimates of test performance may arise. If patients are randomly selected to receive the reference standard the overall diagnostic performance of the test is, in theory, unchanged. In most cases however, this selection is not random, possibly leading to biased estimates of the overall diagnostic accuracy. |
| 6 | Did patients receive the same reference standard regardless of the index test result? | Differential verification bias occurs when some of the index test results are verified by a different reference standard. This is especially a problem if these reference standards differ in their definition of the target condition, for example histopathology of the appendix and natural history for the detection of appendicitis. This usually occurs when patients testing positive on the index test receive a more accurate, often invasive, reference standard than those with a negative test result. The link (correlation) between a particular (negative) test result and being verified by a less accurate reference standard will affect measures of test accuracy in a similar way as for partial verification, but less seriously. |
| 7 | Was the reference standard independent of the index test (i.e. the index test did not form part of the reference standard)? | When the result of the index test is used in establishing the final diagnosis, incorporation bias may occur. This incorporation will probably increase the amount of agreement between index test results and the outcome of the reference standard, and hence overestimate the various measures of diagnostic accuracy. It is important to note that knowledge of the results of the index test alone does not automatically mean that these results are incorporated in the reference standard. For example, a study investigating MRI for the diagnosis of multiple sclerosis could have a reference standard composed of clinical follow-up, CSF analysis and MRI. In this case the index test forms part of the reference standard. If the same study used a reference standard of clinical follow-up and the results of the MRI were known when the clinical diagnosis was made but were not specifically included as part of the reference then the index test does not form part of the reference standard. |
| 8 | Was the execution of the index test described in sufficient detail to permit replication of the test? | A sufficient description of the execution of index test and the reference standard is important for two reasons. Firstly, variation in measures of diagnostic accuracy can sometimes be traced back to differences in the execution of index test or reference standard. Secondly, a clear and detailed description (or citations) is needed to implement a certain test in another setting. If tests are executed in different ways then this would be expected to impact on test performance. The extent to which this would be expected to affect results would depend on the type of test being investigated. |
| 9 | Was the execution of the reference standard described in sufficient detail to permit its replication? | Same above. |
| 10 | Were the index test results interpreted without knowledge of the results of the  reference standard? | This item is similar to "blinding" in intervention studies. Interpretation of the results of the index test may be influenced by knowledge of the results of the reference standard, and vice versa. This is known as review bias, and may lead to inflated measures of diagnostic accuracy. The extent to which this may affect test results will be related to the degree of subjectiveness in the interpretation of the test result. The more subjective the interpretation the more likely that the interpreter can be influenced by the results of the reference standard in interpreting the index test and vice versa. It is therefore important to consider the topic area that you are reviewing and to determine whether the interpretation of the index test or reference standard could be influenced by knowledge of the results of the other test. |
| 11 | Were the reference standard results interpreted without knowledge of the results of the index test? | Same above. |
| 12 | Were the same clinical data available when test results were interpreted as would be available when the test is used in practice? | The availability of clinical data during interpretation of test results may affect estimates of test performance. In this context clinical data is defined broadly to include any information relating to the patient obtained by direct observation such as age, sex and symptoms. The knowledge of such factors can influence the diagnostic test result if the test involves an interpretative component. If clinical data will be available when the test is interpreted in practice then this should also be available when the test is evaluated. If however, the index test is intended to replace other clinical tests then clinical data should not be available, or should be available for all index tests. It is therefore important to determine what information will be available when test results are interpreted in practice before assessing studies for this item. |
| 13 | Were uninterpretable/ intermediate test results reported? | A diagnostic test can produce an uninterpretable/indeterminate/intermediate result with varying frequency depending on the test. These problems are often not reported in diagnostic accuracy studies with the uninterpretable results simply removed from the analysis. This may lead to the biased assessment of the test characteristics. Whether bias will arise depends on the possible correlation between uninterpretable test results and the true disease status. If uninterpretable results occur randomly and are not related to the true disease status of the individual then, in theory, these should not have any effect on test performance. Whatever the cause of uninterpretable results it is important that these are reported so that the impact of these results on test performance can be determined. |
| 14 | Were withdrawals from the study explained? | This occurs when patients withdraw from the study before the results of either or both of the index test and reference standard are known. If patients lost to follow-up differ systematically from those who remain, for whatever reason, then estimates of test performance may be biased. |

Note that these descriptions were cited as they were from the original publication detailing how to apply QUADAS tool in a systematic review.^9^
